# Supplementary material for: HIVprotI: an integrated web based platform for prediction and design of HIV proteins inhibitors
Source: J Cheminform. 2018 Mar 9;10:12. doi: 10.1186/s13321-018-0266-y (PMC5845081; doi:10.1186/s13321-018-0266-y)
Supplement: Supplementary file 2 — Additional file 2. Source code of HIVProtI web server. [file 13321_2018_266_MOESM2_ESM.zip › HIVprotI_Source-code/hivprotI/weka-3-6-6/doc/weka/classifiers/meta/package-summary.html]

weka.classifiers.meta


---


|  |  |  |
| --- | --- | --- |
| **Overview** \| **Package** \| Class \| **Tree** \| **Deprecated** \| **Index** \| **Help** \| **Weka's home** | |  |
| **PREV PACKAGE**   **NEXT PACKAGE** | **FRAMES**    **NO FRAMES**     **All Classes** |  |


---

## Package weka.classifiers.meta

| **Class Summary** | |
| --- | --- |
| **AdaBoostM1** | Class for boosting a nominal class classifier using the Adaboost M1 method. |
| **AdditiveRegression** | Meta classifier that enhances the performance of a regression base classifier. |
| **AttributeSelectedClassifier** | Dimensionality of training and test data is reduced by attribute selection before being passed on to a classifier. |
| **Bagging** | Class for bagging a classifier to reduce variance. |
| **ClassificationViaClustering** | A simple meta-classifier that uses a clusterer for classification. |
| **ClassificationViaRegression** | Class for doing classification using regression methods. |
| **CostSensitiveClassifier** | A metaclassifier that makes its base classifier cost-sensitive. |
| **CVParameterSelection** | Class for performing parameter selection by cross-validation for any classifier.    For more information, see:    R. |
| **Dagging** | This meta classifier creates a number of disjoint, stratified folds out of the data and feeds each chunk of data to a copy of the supplied base classifier. |
| **Decorate** | DECORATE is a meta-learner for building diverse ensembles of classifiers by using specially constructed artificial training examples. |
| **END** | A meta classifier for handling multi-class datasets with 2-class classifiers by building an ensemble of nested dichotomies.    For more info, check    Lin Dong, Eibe Frank, Stefan Kramer: Ensembles of Balanced Nested Dichotomies for Multi-class Problems. |
| **FilteredClassifier** | Class for running an arbitrary classifier on data that has been passed through an arbitrary filter. |
| **Grading** | Implements Grading. |
| **GridSearch** | Performs a grid search of parameter pairs for the a classifier (Y-axis, default is LinearRegression with the "Ridge" parameter) and the PLSFilter (X-axis, "# of Components") and chooses the best pair found for the actual predicting.    The initial grid is worked on with 2-fold CV to determine the values of the parameter pairs for the selected type of evaluation (e.g., accuracy). |
| **LogitBoost** | Class for performing additive logistic regression. |
| **MetaCost** | This metaclassifier makes its base classifier cost-sensitive using the method specified in    Pedro Domingos: MetaCost: A general method for making classifiers cost-sensitive. |
| **MultiBoostAB** | Class for boosting a classifier using the MultiBoosting method.    MultiBoosting is an extension to the highly successful AdaBoost technique for forming decision committees. |
| **MultiClassClassifier** | A metaclassifier for handling multi-class datasets with 2-class classifiers. |
| **MultiScheme** | Class for selecting a classifier from among several using cross validation on the training data or the performance on the training data. |
| **OrdinalClassClassifier** | Meta classifier that allows standard classification algorithms to be applied to ordinal class problems.    For more information see:     Eibe Frank, Mark Hall: A Simple Approach to Ordinal Classification. |
| **RacedIncrementalLogitBoost** | Classifier for incremental learning of large datasets by way of racing logit-boosted committees.    For more information see:    Eibe Frank, Geoffrey Holmes, Richard Kirkby, Mark Hall: Racing committees for large datasets. |
| **RandomCommittee** | Class for building an ensemble of randomizable base classifiers. |
| **RandomSubSpace** | This method constructs a decision tree based classifier that maintains highest accuracy on training data and improves on generalization accuracy as it grows in complexity. |
| **RegressionByDiscretization** | A regression scheme that employs any classifier on a copy of the data that has the class attribute (equal-width) discretized. |
| **RotationForest** | Class for construction a Rotation Forest. |
| **Stacking** | Combines several classifiers using the stacking method. |
| **StackingC** | Implements StackingC (more efficient version of stacking).    For more information, see    A.K. |
| **ThresholdSelector** | A metaclassifier that selecting a mid-point threshold on the probability output by a Classifier. |
| **Vote** | Class for combining classifiers. |

---


|  |  |  |
| --- | --- | --- |
| **Overview** \| **Package** \| Class \| **Tree** \| **Deprecated** \| **Index** \| **Help** \| **Weka's home** | |  |
| **PREV PACKAGE**   **NEXT PACKAGE** | **FRAMES**    **NO FRAMES**     **All Classes** |  |


---
